# Pharmacological and Non-Pharmacological Agents versus Bovine Colostrum Supplementation for the Management of Bone Health Using an Osteoporosis-Induced Rat Model

**DOI:** 10.3390/nu14142837

**Published:** 2022-07-11

**Authors:** Eirini K. Kydonaki, Laura Freitas, Henrique Reguengo, Carlos Raposo Simón, Ana R. Bastos, Emanuel M. Fernandes, Raphaël F. Canadas, Joaquim M. Oliveira, Vitor M. Correlo, Rui L. Reis, Maria Vliora, Paraskevi Gkiata, Yiannis Koutedakis, Georgia Ntina, Rui Pinto, Andres E. Carrillo, Franklim Marques, Tânia Amorim

**Affiliations:** 1 UCIBIO/REQUIMTE, Faculty of Pharmacy, University of Porto, 4050-313 Porto, Portugal; eir.kyd@gmail.com (E.K.K.); laura_c_freitas@hotmail.com (L.F.); hlreguengo@gmail.com (H.R.); franklim@ff.up.pt (F.M.); 2 Centro de Estudios Superiores de la Industria Farmacéutica (CESIF, SA), 28010 Madrid, Spain; craposoc@gmail.com; 3 3B’s Research Group, I3Bs—Research Institute on Biomaterials, Biodegradables and Biomimetics, University of Minho, Headquarters of the European Institute of Excellence on Tissue Engineering and Regenerative Medicine, AvePark, Parque de Ciência e Tecnologia, Zona Industrial da Gandra, 4805-017 Guimarães, Portugal; raquel.bastos@i3bs.uminho.pt (A.R.B.); efernandes@i3bs.uminho.pt (E.M.F.); raphaelcanadas@gmail.com (R.F.C.); miguel.oliveira@i3bs.uminho.pt (J.M.O.); vitorcorrelo@i3bs.uminho.pt (V.M.C.); rgreis@i3bs.uminho.pt (R.L.R.); 4 ICVS/3B’s-PT Government Associate Laboratory, 4805-017 Braga, Portugal; 5 School of Sports and Exercise Sciences, University of Thessaly, 42100 Trikala, Greece; mvliora@gmail.com (M.V.); gkiata.vivi@gmail.com (P.G.); y.koutedakis@uth.gr (Y.K.); 6 Faculty of Education, Health and Wellbeing, University of Wolverhampton, Walsall WS1 3BD, UK; 7 BME, Biomechanical Solutions, 43150 Karditsa, Greece; ntinageorgia10@gmail.com; 8 iMed.UL, Faculty of Pharmacy, University of Lisbon, 1649-003 Lisbon, Portugal; rapinto@ff.ulisboa.pt; 9 JCS, Laboratório de Análises Clínicas Dr. Joaquim Chaves, Avenida General Norton de Matos, 1495-148 Algés, Portugal; 10 Department of Exercise Science, Chatham University, Pittsburgh, PA 15232, USA; acarrillo@chatham.edu; 11 Move-Cor Inc., Pittsburgh, PA 15017, USA

**Keywords:** osteoporosis, alessndronate, vitamin D, calcium, bovine colostrum

## Abstract

Osteoporosis is defined by loss of bone mass and deteriorated bone microarchitecture. The present study compared the effects of available pharmacological and non-pharmacological agents for osteoporosis [alendronate (ALE) and concomitant supplementation of vitamin D (VD) and calcium (Ca)] with the effects of bovine colostrum (BC) supplementation in ovariectomized (OVX) and orchidectomized (ORX) rats. Seven-month-old rats were randomly allocated to: (1) placebo-control, (2) ALE group (7.5 μg/kg of body weight/day/5 times per week), (3) VD/Ca group (VD: 35 μg/kg of body weight/day/5 times per week; Ca: 13 mg/kg of body weight/day/3 times per week), and (4) BC supplementation (OVX: 1.5 g/day/5 times per week; ORX: 2 g/day/5 times per week). Following four months of supplementation, bone microarchitecture, strength and bone markers were evaluated. ALE group demonstrated significantly higher Ct.OV, Ct.BMC, Tb.Th, Tb.OV and Tb.BMC and significantly lower Ct.Pr, Tb.Pr, Tb.Sp, Ct.BMD and Tb.BMD, compared to placebo (*p* < 0.05). BC presented significantly higher Ct.Pr, Ct.BMD, Tb.Pr, Tb.Sp, and Tb.BMD and significantly lower Ct.OV, Ct.BMC, Tb.Th, Tb.OV and Tb.BMC compared to ALE in OVX rats (*p* < 0.05). OVX rats receiving BC experienced a significant increase in serum ALP and OC levels post-supplementation (*p* < 0.05). BC supplementation may induce positive effects on bone metabolism by stimulating bone formation, but appear not to be as effective as ALE.

## 1. Introduction

Osteoporosis is a systemic skeletal disorder defined by loss of bone mass and deteriorated bone microarchitecture [1,2]. This condition is progressive and irreversible, occurring when there is a dysregulation in skeletal remodeling, where bone degradation (mediated by osteoclasts) exceeds bone formation (mediated by osteoblasts). Osteoporosis is the most common disorder of bone metabolism and is considered a serious public health issue as it is associated with several negative outcomes such as increased mortality risk due to osteoporotic fractures [3,4]. According to 2019 estimates, 4.28 million osteoporotic fractures occurred within the European Union (EU) (including Switzerland and United Kingdom; UK) with a direct cost of 36.3 billion euros. By 2024, fracture incidence is expected to increase by 1.06 million in the EU (including the UK and Switzerland) [5]. In the United States (US) it was estimated that 10.2 million of older adults (≥50 years of age) had osteoporosis in 2010 and that figure is expected to increase by more than 30% by 2030 [6]. Globally, it is predicted that the number of people with high risk of osteoporotic fractures (≥50 years of age) will be double by 2040 compared to the estimated 158 million individuals (21 million men and 137 million women) in 2010 [7]. Given that incidents of osteoporosis appear both in younger [8,9] and older individuals [10], prevention and treatment of the condition (and its related fractures) are essential to reduce the associated economic burden and improve patients’ quality of life.

Non-pharmacological management of osteoporosis incorporates recommendations for lifestyle modifications such as exercise and adequate intake of vitamin D (VD) and calcium (Ca) [11,12,13,14]; the concomitant use of calcium and VD supplementation has been recommended for greater benefits [15,16]. Pharmacological agents for the prevention and treatment of osteoporosis include bisphosphonates, receptor activator of nuclear factor kappa-B ligand (RANKL) inhibitor, selective estrogen receptor modulators (SERMs), parathyroid hormone (PTH) analogs, sclerostin inhibitor and hormone replacement therapy (HRT) [17,18]. Although these drugs have been found to be effective in reducing fracture risk, these treatments have also been associated with adverse side effects [17,18]. In fact, there is a low patient adherence to osteoporotic medication due to patients’ concerns of rare side effects, resulting in the exacerbation of the so called “treatment gap” problem [19]. Thus, more attention has been recently placed on natural substances as potential therapeutics for osteoporosis due to minimal side effects [20,21].

Bovine colostrum (BC), the earliest milk produced in the first few days post-partum, is a great candidate as a nutraceutical for osteoporosis as there is evidence in the literature on the beneficial effects of some of its components on bone physiology [22,23,24,25,26], such as lactoferrin (LF) [27,28]. Specifically, BC has been previously suggested as a useful supplement for osteoporosis patients [29,30]. Interestingly, a recently published study using ovariectomized (OVX) and orchidectomized (ORX) rat models for osteoporosis showed that supplementation with BC can improve bone metabolism [31]. Additionally, colostrum was also recently found to reduce the glucocorticoid induced-apoptosis of MC3T3-E1 osteoblasts in vitro [32]. However, to the best of our knowledge, the recently observed positive effects of BC on bone metabolism have not been previously compared with the effects of current established practices for the management and treatment of osteoporosis, such as with the first-line treatment bisphosphonate drug alendronate (ALE). Hence, the aim of the present study was to compare the effects of current available pharmacological and non-pharmacological agents for osteoporosis (ALE and concomitant supplementation of VD and Ca) with the effects of BC supplementation in ovariectomized (OVX) and orchidectomized (ORX) rat models.

## 2. Materials and Methods

### 2.1. Experimental Animal Models

Forty female and thirty-eight male (between seven to fifteen-months old) Wistar Han rats were used in this study. All female rats underwent a bilateral ovariectomy (OVX) and all male rats an orchidectomy (ORX) in order to induce osteoporosis as previously reported [33,34,35]. The surgeries of OVX and ORX were carried out under general anesthesia induced by sequential injections of buprenorphine (0.05 mg/kg body weight, i.p.), metoclopramide (1 mg/kg body weight, i.p.) and a solution of xylazine and ketamine (190 + 100 μL/200 g body weight, i.p.) while maintained with a volatile anesthetic system of 3–4% isoflurane. Post-surgical recovery consisted of isolation from other rats in individual cages for 72 h in a recovery unit station with a temperature of 23 °C and a relative humidity of 45–55% during the first 24 h. During the 72 h of recovery an analgesic plan of orally administered paracetamol (25–400 mg/kg body weight), tramadol (5–20 mg/kg body weight) and metoclopramide (0.2–1 mg/kg) was carried out every 12 h. During the experimental period following recovery, the rats were kept under controlled temperature (22 °C ± 2 °C), relative humidity (55% ± 10%) and 12-h dark/light cycles with an ad libitum access to water and standard rodent feed. Rats were placed in pairs in conventional cages type III and IV, except for four of the older male rats of the study, which were placed in cages individually. At the end of the experimental protocol, the rats were euthanized by an overdose of the same medication used for the surgical anesthesia. All animal procedures were carried out according to the guidelines for animal care and use, while obeying the national and the European Union (EU) directives, specifically the EU directive 2010/63/EU and approved by the National Ethics Committee for the Use of Animals in Research (ORBEA) and by the Ethics Committee of University of Thessaly, Greece (Protocol no. 1351).

### 2.2. Experimental Groups

**Figure 1:**
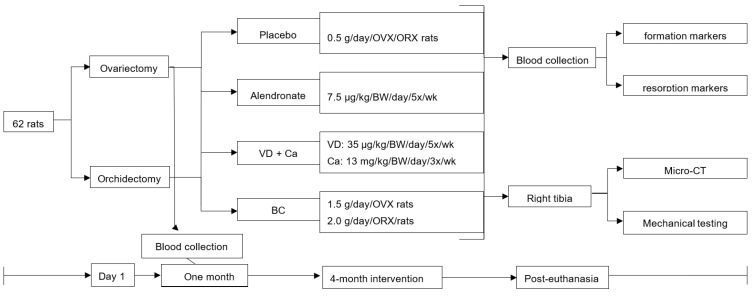
OVX= ovariectomized; ORX = orchidectomized; VD = Vitamin D; Ca = calcium; BC = bovine colostrum; BW = body weight; wk = week.

One month after surgeries, OVX and ORX rats were randomly allocated to 1 of 4 groups with the following treatments: Group (1) placebo-control (OVX, *n* = 8; ORX, *n* = 8): cereal flour-based mash (0.5 g/day), Group (2) ALE (OVX, *n* = 8; ORX, *n* = 8): 7.5 μg/kg of body weight/day/5 times per week, Group (3) VD/Ca (OVX, *n* = 8; ORX, *n* = 6): colecalciferol-35 μg/kg of body weight/day/5 times per week and Ca carbonate–13 mg/kg of body weight/day/3 times per week and Group 4) BC supplementation (OVX, *n* = 8; ORX, *n* = 8): OVX-1.5 g/day/5 times per week; ORX-2 g/day/5 times per week (Figure 1). BC was collected, stored and utilized as previously described [31]. The Ca carbonate (Sandoz) was diluted in distilled water and the ALE was dissolved in a saline vehicle prior to use. The VD (colecalciferol; Merck Serono) did not require preparation prior to use. After the 4-month intervention, blood and bone samples were collected for analysis from euthanized rats. 

### 2.3. Micro-Computed Tomography Analysis

The right tibia of each rat was collected post-euthanasia, placed in small tubes filled with a formaldehyde solution (37%) and stored at 4 °C. Bone microarchitecture of the ORX and OVX rats’ tibias was measured using a high-resolution X-ray microtomography (Micro-CT) system (SkyScan 1272, Kontich, Belgium) as previously described [31]. The different samples were scanned using a pixel size of 4 μm over a rotation range of 360°, a rotation step of 0.45°, and using a 0.25 mm aluminum filter. A standardized cone-beam reconstruction software (NRecon1.6.10.2, Bruker, Kontich, Belgium) was used to reconstruct the 2D cross-sectional images creating a binary picture using at least 30 slices with thresholding between 40 and 255 on a greyscale. Thresholding between 40–60 and 60–255 was applied to differentiate the organic from the inorganic part of the bones, respectively, and quantify the Bone Mineral Content (BMC). The bone mineral density (BMD) was calibrated with the Hounsfield units (HU) using two hydroxyapatite [Ca_10_(PO_4_)_6_(OH)_2_] phantoms with BMD 0.250 and 0.750 g/cm^3^. The 3D morphometric analysis (cortical porosity (Ct.Pr), cortical object volume (Ct.OV), cortical BMD (Ct.BMD), cortical bone mineral content (Ct.BMC), trabecular porosity (Tb.Pr), trabecular separation (Tb.Sp), trabecular thickness (Tb.Th), trabecular object volume (Tb.OV), trabecular BMD (Tb.BMD), and trabecular BMC (Tb.BMC)) were assessed using the CT-analyzer program (CTAn, v1.17.0.0., SkyScan, Belgium).

### 2.4. Biomechanical Testing

Uniaxial tensile tests on the ORX and OVX rats’ tibias were conducted using an Instron 4505 Universal Mechanical Testing Equipment equipped with a BioPlus pneumatic tensile grips system (Instron, MA, USA), as previously mentioned [31]. The tests were performed using a 50 N load cell, and a crosshead speed of 2 mm.min^−1^. The distance between grips was 10 mm. Prior to mechanical tests, the bone specimens were stored in a room at 4 °C and in a formalin solution. Then, the rats’ tibias were removed and washed with distilled water and placed in a phosphate buffered saline (PBS) solution for a period of 2 h before the assays. At least six specimens per condition were tested, including three tibias from rat females and three tibias from males. We determined the maximum tensile strength (σ), which corresponds to the maximum force of the stress–strain curve, and the stiffness of the specimen indicated by the elastic modulus (E). The point of transition between the elastic area and the plastic area of the tensile curve, which is called the yield point, was also determined. This point corresponds to the yield stress (σy) or maximum elastic resistance and also we determined the yield strain (εy), which estimates the capacity of the bone to become strained without suffering micro-fractures.

### 2.5. Bone Biochemical Markers

Following the euthanasia, total circulating blood volume was collected (cardiac, cranial vena cava puncture). Blood was centrifuged to separate the serum which was then stored at −80 °C. The following biomarkers were assessed through ELISA kits: serum osteocalcin (OC, Biorbyt) and alkaline phosphatase (ALP, Mybiosource) for bone formation, and serum deoxypyridinoline (D-Pyr, Mybiosource) for bone resorption [36] according to the manufactures’ protocol. 

### 2.6. Statistical Analyses

Power analysis was performed based on a prior study with a similar design [37]. A required sample of 7 rats per group was indicated by the calculations, assuming a detectable difference of 0.4 standard deviation and 85% power. Statistical analyses conducted to assess differences between treatments and across time were implemented for the OVX and ORX rats independently. Differences in post-intervention measures of bone biochemistry, micro-CT, and biomechanical testing between treatment groups were assessed using a Kruskal-Wallis H test. Post-hoc analyses (Mann-Whitney U) were interpreted using a Bonferroni corrected p value. A Wilcoxon Signed Ranks test was used to determine differences in bone biomarkers assessed across time (pre vs. post treatment). Sex differences (OVX vs. ORX) in all outcome measures were assessed using a Mann-Whitney U test. Statistical analyses were conducted using SPSS (version 27) with differences considered significant at *p* < 0.05. Effect size (d) values were calculated for biomechanical testing and were interpreted as none (0.0–0.19), small (0.2–0.49), medium (0.5–0.79), or large (≥0.8).

## 3. Results

### 3.1. Micro-Computed Tomography Analysis

As expected, OVX rats in the ALE group demonstrated significantly higher Ct.OV, Ct.BMC, Tb.Th, Tb.OV and Tb.BMC, but significantly lower Ct.Pr, Tb.Pr, Tb.Sp compared to placebo (*p* < 0.05) (Table 1). OVX rats supplemented with BC have similar results with the placebo group regarding Ct.Pr, Ct.OV, Ct.BMC, Tb.Pr, Tb.OV and Tb.BMD (*p* > 0.05). However, even though statistical significance was not reached (*p* > 0.0125), OVX rats in the BC group showed lower Ct.BMD, Tb.Sp and Tb.Th and, interestingly, higher Tb.BMC compared to placebo. Comparing OVX rats on ALE with OVX rats on BC supplementation, BC rats had significantly higher Ct.Pr, Ct.BMD, Tb.Pr, Tb.Sp, and Tb.BMD, but significantly lower Ct.OV, Ct.BMC, Tb.Th, Tb.OV and Tb.BMC (*p* < 0.05). OVX rats in the VD/Ca group demonstrated significantly lower Ct.BMD, Tb.Pr, Tb.BMD and Tb.BMC, but significantly higher Tb.OV compared to the placebo group (*p* < 0.05). Further, ORX rats in the ALE group showed significantly lower Ct.BMD and Tb.BMD compared to placebo (*p* < 0.05). ALE administration in ORX rats additionally demonstrated lower Ct.Pr, Tb.Pr, Tb.Sp and higher Ct.OV, Ct.BMC, Tb.Th, Tb.OV was found compared to placebo (not significant, *p* > 0.05). BC ORX group revealed higher Ct.OV, Ct.BMD, Ct.BMC, Tb.Th, Tb.OV, Tb.BMD and Tb.BMC compared to placebo, but lower Ct.Pr, Tb.Pr and Tb.Sp (not significantly, *p* > 0.05). Ct.BMD and Tb.BMD were also found to be significantly higher in the BC group of the ORX rats (*p* < 0.0125) compared to the ALE group. ORX rats in VD/Ca group showed significantly lower Ct.BMD and Tb.BMD compared to placebo (*p* < 0.05).

### 3.2. Biomechanical Testing

Table 2 demonstrates the biomechanical properties of the OVX and ORX rats’ tibias. A large effect of the ALE supplementation was observed for σ (*p* = 0.05, d = 2.17), σy (*p* = 0.05, d = 1.13) and εy (*p* = 0.05, d = 1.13) for the OVX rats; rats on ALE had higher values than the placebo group. Moreover, also in OVX ALE rats, E was found to be lower compared to the placebo group (*p* = 0.05, d = 0.99). In the BC supplementation group of OVX rats, σ (*p* = 0.127, d = 1.06), E (*p* = 0.275, d = 1.32) and σy (*p* = 0.513, d = 0.73) were found to be lower than the placebo, whereas εy (*p* = 0.05, d = 0.75) was found to be higher (than the placebo). OVX rats in the VD/Ca supplementation group revealed similar findings of those on BC supplementation when comparing to the placebo group; i.e., in both VD/Ca and BC groups, σ (*p* = 0.05, d = 1.88), E (*p* = 0.05, d = 3.71) and σy (*p* = 0.05, d = 1.87) were found to be lower than the placebo, whereas εy (*p* = 0.05) was found to be higher (than the placebo). Paradoxically, ORX rats in the ALE group revealed lower σ (*p* = 0.513, d = 0.15), σy (*p* = 0.564, d = 0) and εy (*p* = 0.827, d = 0.36) mechanical values compared to placebo, but higher E (*p* = 0.827, d = 0.19). Further, ORX rats on BC supplementation showed higher σ (*p* = 0.05, d = 3.18), E (*p* = 0.05, d = 2.39), σy (*p* = 0.564, d = 0.69) and lower εy (*p* = 0.827, d = 0.50) compared to the placebo group. ORX rats in the VD/Ca supplementation group had higher mechanical values in all measured properties compared to the placebo group (σ, *p* = 0.275, d = 1.20; E, *p* = 0.05, d = 1.77; σy, *p* = 0.083, d = 2.17; εy, *p* = 0.513, d = 0.25).

### 3.3. Bone Biochemical Markers

OVX rats in the placebo group did not reveal any significant change throughout time in the measured bone biomarkers (*p* > 0.05) (Table 3). However, serum ALP and D-Pyr decreased post-supplementation and OC increased compared to pre-supplementation in the placebo group (*p* > 0.05). Further, a significant decrease of ALP, OC and D-Pyr levels (*p* < 0.05) was seen post-supplementation, though not significantly compared to pre-supplementation in the ALE group (*p* > 0.05). BC OVX rats significantly increased ALP and OC levels post-supplementation (*p* < 0.05). On the contrary, OVX rats in the VD/Ca group significantly decreased ALP and OC levels post-supplementation (*p* < 0.05) (compared to pre-supplementation). Regarding the ORX rats, there was not a difference in ALP, OC and D-Pry levels between pre- and post-supplementation in placebo group (*p* > 0.05). In line with the OVX rats, ORX rats in the ALE group significantly decreased ALP levels post-supplementation (*p* < 0.05). ORX rats in the BC group had similar results to those of OVX rats; i.e., serum ALP and OC levels significantly increased post-supplementation (*p* < 0.05). Serum ALP and OC of ORX rats in the VD/Ca group significantly decreased post-supplementation, whereas D-Pyr significantly increased post-supplementation (*p* < 0.05).

## 4. Discussion

The present study examined the effects of BC supplementation on bone metabolism in comparison with (a) oral administration of ALE and (b) the concomitant supplementation of VD and Ca using an animal osteoporosis model. We showed that BC supplementation positively affected bone metabolism by modulating bone markers; nevertheless, the positive effects of BC on bone mass did not achieve the same level as the positive effects induced by ALE. Concomitant supplementation of VD and Ca did not significantly affect bone metabolism of OVX and ORX rats as compared to the effects of BC or ALE.

Several natural products have been studied for the prevention and/or treatment of osteoporosis [20,38,39]. For instance, oral administration of kefir, a slightly alcoholic and acidic fermented milk, has been found to decrease the levels of C-terminal telopeptides of type I collagen bone turnover marker in OVX rats, similarly to ALE [40]. The oral administration of *Sophora japonica* and *Calligonum comosum* plant extracts have also been shown to improve biochemical markers such as serum Ca, phosphorus, ALP and parathyroid hormone (while increasing Ct.Th and Tb.Th in OVX rats) similarly to ALE administration [41]. Considering BC supplementation, BC has been previously found to reduce osteoblast apoptosis in vitro [32] and to improve the bone metabolism of both OVX and ORX rats [31]. In the latter study, BC induced an improvement in bone microarchitecture as observed by an increase in Ct.BMC and Tb.BMC in OVX rats and an increase in Tb.BMC in ORX rats [31]. BC even appeared to induce the bone formation activity in the ORX rats as seen by an increase of the bone formation biomarker OC [31]. It is believed that the positive effects on bone induced by BC occur mainly due to some of its constituents [23,24,25,26], especially the lactoferrin [22]. Bone anabolic effects of lactoferrin have been well studied and established by several in vitro [28,42,43] and in vivo studies performed in mice [43,44], rats [37,45,46], and in postmenopausal women [47]. Nevertheless, although positive effects of BC on bone metabolism have been reported, the question remains as to whether BC could be used as a supplement to manage osteoporosis. To the best of our knowledge, this is the first study investigating the effects of BC in comparison with ALE (a well-established drug for osteoporosis treatment). Our results support that BC does not induce bone anabolic affects to the same extent as those induced by ALE. Indeed, our study showed that ALE is capable of inducing positive changes in the OVX rats’ microarchitecture as Ct.OV, Ct.BMC, Tb.Th, Tb.OV and Tb.BMC (and lower Ct.Pr, Tb.Pr, and Tb.Sp) in OVX rats under ALE treatment compared to placebo. These findings are in accordance with the mechanical test results performed under tensile load, where positive large effects were found for most of the measured mechanical properties (σ, σy and εy) on the ALE group in comparison to placebo. Moreover, for the ALE treatment, only a slight decrease on the mean value of the bone stiffness (*E*) was notice on the OVX rats. However, the values are in the same range of those obtained in Placebo. Such results highlight the capacity of ALE administration for enhancing intrinsic bone material properties by inducing bone strength and stiffness, being also in accordance with the Micro-CT results. On the other hand, rats under BC supplementation were able to maintain bone parameters at similar levels as the placebo group, but BC was not able to significantly impact OVX rats’ bone mechanical properties. Indeed, only one parameter (εy) was slightly increased in the BC group compared to placebo, while σ, E and the σy were noticeably lower than the placebo. These findings indicate that BC may be capable of inhibiting bone losses and has the potential to exert some bone beneficial effects, but these effects are not as great as the ones induced by ALE. 

Considering bone biomarkers, ALE was found to significantly reduce ALP following the 4-month intervention in the OVX rats. It has been previously reported that ALP and OC increase following ovariectomy, but, after ALE administration, studies have shown that ALP and OC decreases to the same level as prior to ovariectomy [48,49]. Similarly, in the present study we hypothesized that ALP levels increased with ovariectomy, and the ALE treatment reduced ALP levels to the possible normal values the rats had prior to the ovariectomy. This has been considered as a stabilization of bone repair process due to osteoporosis [36,37,41,50,51,52]. Similar to ALE treatment, VD and Ca were expected to induce a significant decrease in the ALP and OC post-supplementation [48], which is in agreement with our data in the OVX rats. BC appeared to significantly increase serum OC and ALP levels in the OVX rats following the 4-month supplementation period, suggesting a positive effect on bone formation.

Regarding ORX rats, the BC group was found to have the most favorable effects on the majority of the bone analyzed parameters evaluated in the current study. Specifically, rats under BC treatment were found to have significantly higher Ct.BMD and Tb.BMD than the ALE and VD/Ca group; and higher Ct.BMD and Tb.BMD values than the placebo group. Rats under BC treatment also exhibited greater mechanical values than the placebo in most of the mechanical bone (σ, E and σy). Moreover, BC supplementation appeared to have potentially stimulated bone formation of the ORX rats as ALP and OC biomarkers were significantly increased post-supplementation. We are not sure why the ALE didn’t improve the bone parameters evaluated in this investigation in the ORX rats as seen with the OVX rats. One hypothesis is that it could have been be due to a potential over-suppression of the bone resorption, a complication previously detected with ALE therapy [53]. If the supplementation period was longer, we would be probably able to observe a higher bone formation rate in ORX rats following alendronate treatment. We suggest though that future studies should increase the time of exposure to alendronate.

BC supplementation has been used in several therapeutic applications such as gastrointestinal disorders, allergies and autoimmune diseases, viral and bacterial illnesses, and HIV-associated immunomodulation HIV. However, to our knowledge, the possibility of using a nutraceutical supplementation based on bovine colostrum to reduce bone losses has not been considered. According to the present study, BC does not induce positive changes on bone health to the same extent as ALE in rats with osteoporosis. However, considering previous reports on the effects of BC on bone parameters and the positive effects of BC shown in the present study, the results suggest that BC supplementation may be effective in maintaining bone health or delaying the onset of osteoporosis. Future studies should test the effects of BC in other populations as healthy individuals, and pre- and post-menopausal.

There are strengths and limitations to the present study. To our knowledge, the present study is the first to investigate whether BC, a natural supplementation, could induce the same effects in bone metabolism as well-established drugs for osteoporosis. Considering the increased public interest on using natural supplements, we believe our study is relevant. Another strength of the present study is the inclusion of both OVX and ORX animal models for osteoporosis management, given that most relevant literature only focuses on females. Nevertheless, it is reasonable to assume that the present study might have been influenced by methodological limitations as well, such as the absence of BC component analyses. Further, even though the number of rats in each group was determined by power calculations, the analyses of the mechanical test were performed in only a few individuals per group. Another limitation is the absence of body weight and food intake monitoring data throughout the intervention. It is well known that diet and body weight interfere with bone health outcomes. Future studies should assess body weight before and after supplementation. Moreover, we acknowledge that the number of rats per group is rather small. Future studies should consider increasing the sample size and include a healthy control group to validate the present study’s preliminary results. Indeed, by including a healthy control group it would be possible to examine if the results obtained from the BC supplementation, following induced osteoporosis, would be comparable to those of the healthy control group.

In conclusion, BC supplementation has the potential to improve the bone physiology of OVX and ORX rats by stimulating bone formation, however not to the degree of current drug treatment ALE for osteoporosis.

## Figures and Tables

**Table 1:** Post-supplementation bone microarchitecture results of the placebo, alendronate, vitamin D and calcium and BC supplementation groups–OVX and ORX rats.

	Post-Intervention			
Analyzed Parameter	Placebo (OVX, ORX: *n* = 8)	Alendronate (OVX, ORX: *n* = 8)	Vit. D + Calcium (OVX: *n* = 8; ORX: *n* = 6)	BC (OVX, ORX: *n* = 8)
Cortical bone				
Porosity (%)				
OVX rats	26.6 ± 11.1 ^a^	10.8 ± 1.5 ^b^	23.2 ± 14.4 ^a^	25.2 ± 8.5 ^a^
ORX rats	29.5 ± 4.2	21.5 ± 13.6	28.5 ± 15.0	25.9 ± 7.4
Volume (% BV/TV)				
OVX rats	73.4 ± 11.1 ^a^	89.2 ± 1.5 ^b^	76.8 ± 14.4 ^a,b^	74.8 ± 8.5 ^a^
ORX rats	70.4 ± 4.1	78.6 ± 13.6	71.5 ± 15.0	74.1 ± 7.4
BMD (g/cm^3^)				
OVX rats	2.93 ± 0.29 ^a^	0.92 ± 0.09 ^b^	1.05 ± 0.15 ^b^	2.83 ± 0.31 ^a^
ORX rats	2.84 ± 0.33 ^a^	1.67 ± 0.60 ^b^	1.07 ± 0.07 ^b^	2.93 ± 0.37 ^a^
BMC (g)				
OVX rats	71.2 ± 9.6 ^a^	91.9 ± 5.0 ^b^	58.8 ± 31.4 ^a,b^	71.8 ± 14.8 ^a^
ORX rats	72.0 ± 12.9	73.5 ± 17.5	44.8 ± 31.6	78.9 ± 10.9
Trabecular bone				
Porosity (%)				
OVX rats	87.2 ± 2.0 ^a^	78.6 ± 3.6 ^b^	73.9 ± 7.8 ^b^	85.3 ± 2.2 ^a^
ORX rats	87.2 ± 4.1	84.8 ± 5.7	84.0 ± 7.5	84.9 ± 4.2
Separation (µm)				
OVX rats	163.1 ± 86.6 ^a^	46.2 ± 7.8 ^b^	82.9 ± 61.7 ^a,b^	104.6 ± 47.4 ^a^
ORX rats	113.0 ± 96.6	104.9 ± 54.6	141.1 ± 98.8	77.9 ± 22.4
Thickness (µm)				
OVX rats	25.7 ± 20.5 ^a^	41.6 ± 12.7 ^b^	21.6 ± 5.0 ^a^	18.4 ± 2.5 ^a^
ORX rats	16.4 ± 1.4	33.1 ± 18.5	20.3 ± 6.0	16.8 ± 1.6
Volume (% BV/TV)				
OVX rats	12.8 ± 2.0 ^a^	21.4 ± 3.6 ^b^	26.1 ± 7.8 ^b^	14.7 ± 2.2 ^a^
ORX rats	12.8 ± 4.1	15.2 ± 5.7	16.0 ± 7.5	15.1 ± 4.2
BMD (g/cm^3^)				
OVX rats	1.20 ± 0.12 ^a^	−0.02 ± 0.05 ^b^	0.03 ± 0.04 ^b^	1.22 ± 0.12 ^a^
ORX rats	1.23 ± 0.20 ^a^	0.04 ± 0.04 ^b^	0.02 ± 0.03 ^b^	1.27 ± 0.25 ^a^
BMC (g)				
OVX rats	61.2 ± 10.2 ^a^	78.2 ± 4.7 ^b^	56.1 ± 26.4 ^b^	72.7 ± 13.4 ^a,b^
ORX rats	64.8 ± 12.9	59.4 ± 13.8	40.8 ± 30.9	74.2 ± 10.2

QCT analysis were made in the right posterior limb. Values are mean ± SD. Non-parametric tests were used to compare groups. Statistical significance was set at 0.0125. ^a,b^ Means in the same row with different lowercase superscript letters are significantly different (*p* < 0.0125). BMD = bone mineral density; BMC = bone mineral content.

**Table 2:** Mechanical properties of the OVX and ORX rats’ tibias.

	Post-Intervention			
Analyzed Parameter	Placebo (OVX, ORX: *n* = 3)	Alendronate (OVX, ORX: *n* = 3)	Vit. D + Calcium (OVX, ORX: *n* = 3)	BC (OVX, ORX: *n* = 3)
Max. tensile strength (σ, MPa)				
OVX rats	8.0 ± 0.8	11.4 ± 1.6	6.4 ± 0.6	6.2 ± 1.7
ORX rats	3.8 ± 0.6	3.4 ± 2.9	8.0 ± 3.9	6.0 ± 0.5
Elastic modulus (E, MPa)				
OVX rats	385.1 ± 54.1	336.4 ± 11.4	201.1 ± 14.3	277.4 ± 74.1
ORX rats	151.8 ± 35.3	162.9 ± 54.4	270.4 ± 66.7	239.1 ± 21.4
Stress at yield (σy, MPa)				
OVX rats	3.5 ± 0.5	5.3 ± 1.7	2.7 ± 0.2	3.1 ± 0.5
ORX rats	1.7 ± 0.3	1.7 ± 0.5	4.2 ± 1.0	2.5 ± 1.0
Strain at yield (εy, %)				
OVX rats	1.1 ± 0.0	1.7 ± 0.6	1.5 ± 0.0	1.3 ± 0.3
ORX rats	1.4 ± 0.2	1.2 ± 0.6	1.5 ± 0.4	1.2 ± 0.4

Uniaxial tensile tests were made in the right posterior limb. Values are mean ± SD. Non-parametric tests were used to compare groups. Statistical significance was set at 0.0125. Max. = maximum.

**Table 3:** Bone biomarkers–evaluation across time (pre-post intervention) of the placebo, alendronate, vitamin D and calcium and BC supplementation groups–OVX and ORX rats.

Analyzed Parameter	Placebo (OVX, ORX: *n* = 8)	Alendronate (OVX, ORX: *n* = 8)	Vit. D + Calcium (OVX: *n* = 8; ORX: *n* = 6)	BC (OVX, ORX: *n* = 8)
Alkaline phosphatase (U/L)				
OVX rats				
Pre	98.8 ± 12.2 ^b^	205.1 ± 287.0 ^b^	59.9 ± 13.7 ^a^	67.1 ± 19.6 ^a^
Post	92.1 ± 26.3 ^a^	93.2 ± 8.6 ^a,^*	17.1 ± 4.9 ^b,^*	70.8 ± 19.9 ^a,^*
ORX rats				
Pre	114.4 ± 11.2 ^a^	100.3 ± 8.5 ^a^	59.0 ± 13.0 ^b^	106.8 ± 19.9 ^a^
Post	114.5 ± 10.7 ^a^	93.9 ± 7.4 ^b,^*	18.0 ± 3.6 ^c,^*	119.7 ± 21.9 ^a,b,^*
Osteocalcin (µg/L)				
OVX rats				
Pre	11.9 ± 1.7 ^a^	12.4 ± 1.9 ^a^	8.1 ± 0.7 ^b^	12.2 ± 2.3 ^a^
Post	13.4 ± 2.5 ^a,b^	12.2 ± 1.7 ^b^	7.5 ± 0.4 ^c,^*	15.6 ± 2.2 ^a,^*
ORX rats				
Pre	10.6 ± 0.8 ^b,c^	11.5 ± 1.8 ^b^	9.1 ± 1.5 ^c^	14.8 ± 1.9 ^a^
Post	10.7 ± 0.6 ^b^	11.4 ± 1.7 ^b^	7.5 ± 0.4 ^c,^*	16.6 ± 1.5 ^a,^*
Deoxypyridinoline (µg/L)				
OVX rats				
Pre	0.44 ± 0.18	0.38 ± 0.10	0.46 ± 0.11	0.34 ± 0.1
Post	0.43 ± 0.16	0.37 ± 0.06	0.47 ± 0.08	0.34 ± 0.1
ORX rats				
Pre	0.43 ± 0.06 ^a^	0.28 ± 0.06 ^b^	0.42 ± 0.09 ^a^	0.37 ± 0.11 ^a,b^
Post	0.44 ± 0.04 ^a^	0.27 ± 0.05 ^b^	0.52 ± 0.08 ^a,^*	0.37 ± 0.11 ^a,b^

Values are mean ± SD. Non-parametric tests were used to compare groups and within each group pre-post evaluation. Statistical significance was set at 0.0125. ^a–c^ Means in the same row with different lowercase superscript letters are significantly different (*p* < 0.0125). * *p* < 0.05 significant different pre-post evaluation within the same group.

## Data Availability

The datasets generated during and/or analyzed during the current study are available from the corresponding author upon a reasonable request.
